# Spatiotemporal Clustering of Repeated Super-Resolution Localizations via Linear Assignment Problem

**DOI:** 10.3389/fbinf.2021.724325

**Published:** 2021-10-20

**Authors:** David J. Schodt, Keith A. Lidke

**Affiliations:** Department of Physics and Astronomy, University of New Mexico, Albuquerque, NM, United States

**Keywords:** fluorescence microscopy, super-resolution, image analysis, computational modeling, single molecule techniques

## Abstract

Many fluorescence super-resolution techniques, such as (d)STORM, PALM, and DNA-PAINT, generate datasets wherein multiple localizations across many camera frames may arise from a single blinking event of an emitter. These repeated localizations not only hinder interpretation and analysis of such datasets, but also represent an incomplete use of the fluorescence photons. Such localizations are typically combined into a single localization either by clustering with hard distance and time thresholds, or by classical hypothesis testing assuming Gaussian localization errors. In this work, we describe a method for clustering that accounts for localization precision, local emitter density estimates, and a kinetic model for blinking which is used to optimize connections within a group of spatiotemporally colocated localizations.

## 1 Introduction

Fluorescence super-resolution methods have grown to be vital imaging techniques in many research areas, particularly in the biological sciences. Single Molecule Localization Microscopy (SMLM) methods take advantage of an extreme form of temporal independence where individual sources blink on and off with little spatial-temporal overlap from other “on” sources. Many of these techniques, such as (d)STORM (Rust et al., 2006; Heilemann et al., 2008), PALM (Betzig et al., 2006; Hess et al., 2006), and DNA-PAINT (Jungmann et al., 2010), are relatively easy to implement on common fluorescence microscopes with little to no modifications. By finding the center of distinct PSFs arising from independent “on” sources as observed on a camera, SMLM data is reduced to a set of PSF center coordinates, or localizations, and their associated precisions. The subsequent processing of these localizations can have significant impacts on the final interpretation of the data.

Despite extensive research into optimally localizing emitters (Small and Stahlheber, 2014; Deschout et al., 2014; Sage et al., 2019), little effort has been spent on what we will henceforth refer to as the frame-connection problem. SMLM methods produce data with multiple localizations in subsequent/near-subsequent frames which are likely the result of a single blinking event of a single emitter. Specifically, a single visible emitter may appear in multiple frames, with each frame potentially producing a new localization of that emitter. The frame-connection problem deals with combining these repeated localizations into a single localization with higher precision. To the best of our knowledge, only two solutions to the frame-connection problem are in use: 1) combining any localizations within *N* frames and *d* pixels of one another, as is done in the popular ThunderSTORM package (Ovesný et al., 2014) (we’ll refer to this method as the “classical” approach); or 2) by a hypothesis test assuming Gaussian localization noise (“hypothesis test”) (Wester et al., 2021). A modification to the classical approach involves setting the separation threshold *d* to be some multiple of the localization error, as is done in the PYMEVisualize package (Marin et al., 2021) (referred to as “chaining” in that work and as “revised classical” here). The classical approach has the benefit of simplicity: its implementation is straightforward and accessible. The hypothesis testing approach makes use of localization error to test the null hypothesis that localizations came from the same emitter, however it neglects a calculation and comparison to the low-probability alternative hypothesis that a new emitter could have appeared in the same location. Both of these methods implicitly make use of the prior knowledge that multiple blinking events within a small spatiotemporal volume is rare in SMLM data.

To ensure information about the underlying structure of emitters is retained, an optimal frame-connection solution should exhibit minimal over-clustering. In other words, a frame-connection algorithm prone to connecting localizations from distinct emitters may reduce the quality of SMLM data. However, if a frame-connection algorithm is prone to under-clustering localizations from a single blinking event, it’s addition to the analysis pipeline may not represent much value. In that view, an optimal frame-connection solution must be capable of clustering those localizations which are very likely to have arisen from a single blinking event of a single emitter, all the while remaining sufficiently conservative in its connection assignments to minimize over-clustering.

The analysis of SMLM localizations can be classified into two categories: pre- and post-processing. Broadly speaking, pre-processing is a clean-up stage during which raw localizations are filtered without destroying the information they carry. Frame-connection should be considered pre-processing in the sense that its goal is to combine repeated localizations without destroying the temporal information carried by emitter blinking. In contrast, post-processing methods aim to condense/summarize the information carried by the localizations into descriptors of the underlying structure or process being observed. More general post-processing clustering methods, such as DBSCAN (Daszykowski et al., 2001), Voronoi tesselation (Levet et al., 2015), and BaGoL (Fazel et al., 2019b), differ from pre-clustering frame-connection in that they are not restricted to grouping observations of a single blinking event. Rather, post-processing clustering methods attempt to associate or make inference from all localizations of a single emitter.

The analysis of single-particle tracking (SPT) data aims to achieve a similar goal to frame-connection: associating multiple localizations over time to a single emitter. The ideal solution to the SPT problem is global across all connection possibilities; however, such a solution is not computationally feasible for realistic experiments. A locally greedy solution is prone to incorrect/missed connections, a problem exacerbated by emitter blinking and detection failure. As a result, many SPT analysis methods approximate a global solution by performing a locally greedy (in time) step to reduce the computational complexity. For example, the multiple target tracing (MTT) method (Sergé et al., 2008) considers only those connection hypotheses corresponding to a sliding spatiotemporal window. The method presented in (Jaqaman et al., 2008) performs an initial frame-to-frame connection followed by a global gap closing procedure.

In this work, we present a novel solution to the single blinking event frame-connection problem which accounts for local emitter densities, fluorescent emission kinetics, and localizations missed in processing, which we refer to as linear assignment problem frame-connection (LAP-FC). Motivated by the success and robustness of the cost matrix method to solving the linear assignment problem (LAP) in SPT (Jaqaman et al., 2008), we formulate the frame-connection problem in terms of the costs of connecting/not connecting localizations. Our algorithm effectively groups all reasonable connection hypotheses in a pre-processing step (enabled by the typical brevity of blinking events in SMLM data), which allows us to find a globally optimal solution to the single blinking event frame-connection problem. We demonstrate that our algorithm outperforms the classical and hypothesis test methods in several situations typical of SMLM data with no to minimal evidence of over-clustering. Furthermore, our algorithm is in practice parameter-free, making it the ideal method for use by end users of SMLM data.

## 2 Materials and Methods

Our solution to the frame-connection problem consists of three primary components: 1) pre-clustering of localizations into sets of connection candidates, 2) estimating local densities and kinetic rates from preclusters, and 3) making a maximum likelihood assignment of localizations to clusters, which is implemented as a LAP. In this section, we will describe our formulation of the frame-connection problem before describing the three components of our algorithm. A description of some commonly used variables used throughout this text is provided in Table 1.

**TABLE 1:** Description of commonly used variables.

Variable	Description	Units
*k*_on_	transition rate from the emitter dark state to the on (visible) state	frame^−1^
*k*_off_	transition rate from the emitter on state to the reversible off state	frame^−1^
*k*_bleach_	transition rate from the emitter on state to the irreversible bleached state	frame^−1^
*p*_miss_	probability of failing to localize a visible emitter	
*n*	total number of (pre-frame connection) localizations in the data	
*n*_*c*_	number of localizations in a given precluster	
*N*_emitters_	underlying number of emitters in the data	
*ρ*_0_	initial underlying density of emitters in the first frame of data	emitters/pixel^2^
*ρ*	underlying density of non-bleached emitters	emitters/pixel^2^
*ρ*_on_	density of emitters in the “on” state	emitters/pixel^2^
*ρ*_off_	density of emitters in the “off” state	emitters/pixel^2^
*N*	number of spatial dimensions	
**x**	vector of Cartesian coordinates [*x*_1_, *x*_2_, … , *x*_*N*_]	pixels
Δ*x*_*i*_	separation between two localizations along the *i*-th dimension	pixels
variance of the first localization in the *i*-th dimension	pixels^2^
variance of the second localization in the *i*-th dimension	pixels^2^
$\sigma_{x_{i}}^{2}$	sum of the variances $\sigma_{x_{i},1}^{2}+\sigma_{x_{i},2}^{2}$	pixels^2^
*f*	integer frame number	frames
*f*_end_	frame number corresponding to the last frame of the data	frames
*N*_*p*_	number of candidate frames that have elapsed by the appearance of a localization	frames
*N*_*f*_	number of candidate frames remaining after the appearance of a localization	frames
*τ*	approximate duty cycle of an emitter	
*F*	CDF of the nearest-neighbor distribution of localizations within 5 frames of one another	
*δ*	deviation of a nearest-neighbor distribution CDF *F* from the ideal CDF *F*_ideal_	

### 2.1 Pre-Clustering

For a typical SMLM dataset, the number of localizations *n* ∼ 10^6^ makes finding a global solution to the LAP across all localizations computationally infeasible. As such, we perform a pre-clustering of localizations in a manner similar to the revised classical frame-connection solution as presented in (Marin et al., 2021). For a given localization, the spatial nearest neighbor within some frame gap and within some multiplier of its localization error (typically chosen to be five frames and 5, respectively) is found. If that nearest neighbor is already part of a cluster, the localization is incorporated into that same cluster. Otherwise, the localization and its nearest neighbor (if one exists) are defined as a new cluster. To ensure localizations aren’t excluded from their ideal precluster, pre-clustering allows incorporation of multiple localizations within the same frame to the same cluster.

### 2.2 Estimating Local Emitter Densities and Kinetic Rates

To estimate local emitter densities and kinetic rates, we assume that each precluster will on average be representative of a single blinking event. That is, we assume that most preclusters consists of localizations of a single emitter blinking once with a duration of multiple frames. The rate parameters *k*_on_, *k*_off_, and *k*_bleach_, and the probability of missing a localization *p*_miss_ are estimated from the pre-clustered data as follows. The sum of the off rate and the bleaching rate *k*_off_ + *k*_bleach_ is estimated from the cluster durations *N* (in frames) as

$$\hat{k_{\text{off}}+k_{\text{bleach}}}=-\log\left(1-\frac{1}{\bar{N}}\right)$$ (1)

where $\bar{...}$ denotes the mean value. The expression given in Eq. 1 is derived assuming that cluster durations are geometrically distributed with the probability of turning off given by 1 − exp[− (*k*_off_ + *k*_bleach_)] (the probability of turning off within Δ*t* = 1 frames). The probability of missing a localization *p*_miss_ is estimated from the ratio of the number of localizations in a cluster *n*_*c*_ to the cluster’s duration *N* as

$${\hat{p}}_{\text{miss}}=1-\bar{n_{c}/N}$$

The expected cumulative number of localizations observed by frame *f* is given by

$$\left\langle n_{\text{cumulative}}\right\rangle \left(f\right)\approx N_{\text{emitters}}\left(1-p_{\text{miss}}\right)\tau \left\{\frac{1-\exp\left[-\lambda_{1}\left(f-1\right)\right]}{\lambda_{1}}-\frac{1-\exp\left[-\lambda_{2}\left(f-1\right)\right]}{\lambda_{2}}\right\}$$ (2)

with

$$\begin{array}{ll} \lambda_{1} & =k_{\text{bleach}}\frac{k_{\text{on}}}{k_{\text{on}}+k_{\text{off}}+k_{\text{bleach}}}\equiv k_{\text{bleach}}\tau \\ \lambda_{2} & =k_{\text{on}}+k_{\text{off}}+k_{\text{bleach}}-\lambda_{1} \end{array}$$

where *N*_emitters_ is the total number of emitters present at the beginning of the experiment. Eq. 2 was derived from the results presented in (Nino and Milstein, 2021) by assuming *k*_on_ ≪ *k*_off_ with no restriction on *k*_bleach_ and by accounting for *p*_miss_. Similarly, the cumulative number of preclusters observed over time is of the form

$$\langle n_{\text{clusters cumulative}}\rangle \left(f\right)\approx k_{\text{off}}\langle n_{\text{cumulative}}\rangle \left(f\right)$$ (3)

According to Eqs. 2, 3, the off rate *k*_off_ can be estimated as *n*_clusters_/*n* where *n*_clusters_ is the total number of preclusters and *n* is the total number of localizations. The bleaching rate *k*_bleach_ is then found by subtracting the estimate for *k*_off_ from Eq. 1. The on rate *k*_on_ and the underlying number of emitters *N*_emitters_ are then estimated by fitting the cumulative number of localizations to the model given in Eq. 2. Additional details about the parameter estimation procedures can be found in Supplementary Text 1.

The local pre-cluster density corresponding to each pre-cluster is estimated by finding the *k* (chosen to be two in this study) nearest pre-clusters and then computing $\rho_{c}=\left(k+1\right)/\pi r_{k}^{2}$ where *r*_*k*_ is the distance to the *k*th nearest pre-cluster. The underlying local emitter density present at the beginning of the experiment is then estimated for each pre-cluster as

$${\hat{\rho }}_{0,\text{local}}=\rho_{c}\frac{1}{{\hat{k}}_{\text{off}}\hat{\tau }}\frac{1}{1-{\hat{p}}_{\text{miss}}}{\left\{\frac{1-\exp\left[-{\hat{\lambda }}_{1}\left(f_{\text{end}}-1\right)\right]}{{\hat{\lambda }}_{1}}-\frac{1-\exp\left[-{\hat{\lambda }}_{2}\left(f_{\text{end}}-1\right)\right]}{{\hat{\lambda }}_{2}}\right\}}^{-1}$$

where *f*_end_ is the last frame containing localizations in the experiment. The density of on emitters *ρ*_on_ and the density of off emitters *ρ*_off_ are then estimated as

$$\begin{array}{ll} {\hat{\rho }}_{\text{on}}\left(f\right) & ={\hat{\rho }}_{0,\text{local}}\hat{\tau }\left\{\exp\left[-{\hat{\lambda }}_{1}\left(f-1\right)\right]-\exp\left[-{\hat{\lambda }}_{2}\left(f-1\right)\right]\right\}\\ {\hat{\rho }}_{\text{off}}\left(f\right) & ={\hat{\rho }}_{\text{on}}\left(f\right)\frac{{\hat{k}}_{\text{off}}}{{\hat{k}}_{\text{on}}} \end{array}$$

### 2.3 Frame-Connection via Minimization of Costs

The frame-connection problem can be thought of as a spatiotemporal clustering problem in which only one localization is allowed admittance to each cluster in each frame. In terms of the LAP, frame-connection concerns assigning each observed localization to one and only one cluster, with each assignment having an associated cost. In particular, frame-connection consists of “connection” costs, “birth” costs, and “death” costs. The connection costs are the costs for assigning a localization to an existing cluster. The birth costs are the costs for birthing a new emitter with the candidate localization being its first observation. The death costs are the costs for prohibiting assignment of any future localizations to an existing localization cluster. The costs are arranged in a square matrix such that the LAP solution permits only one assignment per row and column. We define each of these costs by assuming a three-state kinetic model for emitter blinking. The transition rates are defined as *k*_on_, the rate from the (reversible) off state to the visible on state; *k*_off_, the rate from the on state to the off state; and *k*_bleach_, the rate from the on state to the (irreversible) bleached state. We additionally assume a constant probability of missing a localization (i.e., failing to localize a visible emitter) which we designate *p*_miss_. Furthermore, the costs account for the local density of emitters *ρ*(**x**, *f*) where **x** = [*x*, *y*] is the precluster location and *f* is the frame number. Our procedure for estimating *k*_on_, *k*_off_, *k*_bleach_, *p*_miss_, and *ρ*(**x**, *f*) directly from the data is described in section 2.2.

The connection, birth, and death costs are defined to be the negative logarithm of the probabilities associated with the prescribed actions. The cost *c*_*c*_ of connecting two localizations is defined as follows:

$$c_{c}=-\log\left\{\overset{N}{\underset{i=1}{\prod }}p\left(\Delta x_{i}|\sigma_{x_{i}}^{2}\right)\cdot p\left(\text{observe after missing localizations}|p_{\text{miss}},\Delta f\right)\cdot p\left(\text{not turning off}|\Delta f\right)\right\}$$

where *N* is the number of dimensions (taken to be 2 for the present study), Δ*x*_*i*_ is the separation between the two localizations along the *i*-th dimension, $\sigma_{x_{i}}^{2}\equiv \sigma_{x_{i},1}^{2}+\sigma_{x_{i},2}^{2}$ is the sum of the localization variances $\sigma_{x_{i},1}^{2}$ for localization 1 and $\sigma_{x_{i},2}^{2}$ for localization 2 in the *i*-th dimension, and Δ*f* > 0 is the temporal separation between the two localizations. The probability terms are given by

$$\begin{array}{ll} & p\left(\Delta x_{i}|\sigma_{x_{i}}^{2}\right)=\frac{1}{\sqrt{2\pi \sigma_{x_{i}}^{2}}}\exp\left(\frac{\Delta x_{i}^{2}}{2\sigma_{x_{i}}^{2}}\right)\\ & p\left(\text{observe after missing localizations}|p_{\text{miss}},\Delta f\right)=\left(1-p_{\text{miss}}\right)p_{\text{miss}}^{\Delta f-1}\\ & p\left(\text{not turning off}|\Delta f\right)=\exp\left[-\left(k_{\text{off}}+k_{\text{bleach}}\right)\Delta f\right] \end{array}$$

where the rate parameters are given in units of frame^−1^. The cost of introducing a new emitter in frame *M* after *N*_*p*_ candidate frames (“birth” cost) is given by

$$\begin{array}{ll} c_{b}=-\log & \left\{p\left(\text{new emitter turning on}|k_{\text{on}},\rho_{\text{off}}\left(x,f\right),N_{p}\right)\cdot p\left(\text{not missing localization}|p_{\text{miss}}\right)\right.\\ + & \left.p\left(\text{observe after missing localizations}|p_{\text{miss}},N_{p},\rho_{\text{on}}\left(x,f\right)\right)\right\}\\ =-\log & \left\{\rho_{\text{off}}\left(x,M\right)\left[1-\exp\left(-k_{\text{on}}\right)\right]\exp\left(-k_{\text{on}}N_{p}\right)\left(1-p_{\text{miss}}\right)\right.\\ + & \left.\rho_{\text{on}}\left(x,M-N_{p}\right)\left(1-p_{\text{miss}}\right)p_{\text{miss}}^{N_{p}}\right\} \end{array}$$

where *ρ*_off_ (**x**, *f*) is the local density of emitters in the off state, and *ρ*_on_ (**x**, *f*) is the local density of emitters in the on state (see section 2.2). The cost of not observing an emitter for the remaining *N*_*f*_ candidate frames (“death” cost) is given by

$$\begin{array}{ll} c_{d} & =-\log\left\{p\left(\text{bleaching}|k_{\text{bleach}}\right)+p\left(\text{turn off}|k_{\text{off}}\right)+p\left(\text{missing localizations}|p_{\text{miss}},N_{f}\right)\right\}\\ & =-\log\left\{\left[1-\exp\left(-k_{\text{bleach}}\right)\right]+\left[1-\exp\left(-k_{\text{off}}\right)\right]+p_{\text{miss}}^{N_{f}}\right\} \end{array}$$

As in (Jaqaman et al., 2008), we arrange our LAP costs in a square block matrix composed of four equal sized square sub-matrices, with each sub-matrix being *n*_*c*_ × *n*_*c*_ for *n*_*c*_ localizations within a given precluster. The upper-left block matrix contains the connection costs between a localization identified by its row index with a localization identified by its column index, arranged as an upper-triangular matrix (to prohibit selection pairs of row m to column n and row n to column m) and divided by two to account for the definition of the bottom-right auxiliary block costs (see below). The bottom-left block contains the birth costs for the localizations identified by the column index. The upper-right block contains the death costs for the localizations identified by the row index. The bottom-right block, to which we attribute no physical significance, is defined to be the transpose of the upper-left connection block, as assignments in the upper-left block lead to the same assignments in the (transposed) lower-right block. All cost matrix entries containing a prohibited selection (e.g., main diagonal terms, which represent connection of a localization to itself) are set to a non-link marker, which tells the LAP solver not to select those entries. Costs that are infinite or otherwise invalid (i.e., not a number) are set to twice the sum of all valid costs to ensure they are only selected when no other assignment is available. The LAP is then solved using the Jonker-Volgenant algorithm (Jonker and Volgenant, 1987), which assigns each localization to a single cluster of localizations. This process is then repeated for each pre-cluster of localizations to yield the final frame-connected set of localizations.

Localizations connected by the frame-connection algorithm are combined assuming they each represent independent samples from a Gaussian distribution. The resulting position of the *m* frame-connected localizations is taken to be the maximum-likelihood estimate for the position **x**

$$\hat{x}=\frac{\sum_{i=1}^{m}x_{i}/\sigma_{i}^{2}}{\sum_{i=1}^{m}1/\sigma_{i}^{2}}$$ (4)

and the localization error for the frame-connected localization is taken to be the inverse of the Fisher information

$${\hat{\sigma }}^{2}=\frac{1}{\sum_{i=1}^{m}1/\sigma_{i}^{2}}$$ (5)

### 2.4 Simulated SMLM Data

Simulated SMLM localizations were generated to test the frame-connection algorithms. A uniformly distributed point target was simulated by scattering emitters uniformly across a square region of interest (ROI). Dimerized emitters were simulated by placing emitters at varying separations from one another with sufficient inter-dimer spacing to ensure localizations from distinct dimers will not be connected by any of the algorithms. To generate localizations from simulated emitter positions, the frames in which each emitter was observed were simulated by the Gillespie algorithm (Gillespie, 1976) as prescribed by the transition rates *k*_on_ = 0.005 frame^−1^, *k*_off_ = 0.5 frame^−1^, and *k*_bleach_ = 0.2 frame^−1^. Localizations corresponding to the emitter being on the entire frame were assigned a fixed photon count *I*. For frames in which the emitter turned on, turned off, or bleached, the number of photons was reduced to *I* (1 − *u*) where *u* is sampled from the standard uniform distribution. Gaussian noise was added to each localization with a standard deviation given by the Cramér-Rao lower bound corresponding to fitting a Gaussian to the emitter PSF given the background intensity and a finite pixel size (Smith et al., 2010). To mimic noise sources not accounted for by localization errors, such as residual, uncorrected sample drift, an additional source of Gaussian noise with standard deviation 0.05 pixels was added to each localization. A constant probability of missing a localization *p*_miss_ was applied to the final results by randomly removing *p*_miss_ *n* (rounded to the nearest integer) localizations from the *n* total localizations.

### 2.5 Comparison to “Ideal” Results

The “ideal” frame-connection results are specified as follows. For a given simulation, the underlying emitter generating each localization is noted. Localizations arising from the same emitter that occur within five frames of one another are then combined using Eqs. 4, 5. These frame-connected localizations are considered to be the “ideal” frame-connection result.

The cumulative distribution function (CDF) of the nearest-neighbor distance distribution between frame-connected localizations was computed as follows. For a given set of frame-connected localizations, the nearest-neighbor to each localization within five frames (in the past or into the future, but excluding same frame neighbors) was found and their separation was stored. The binned CDF was then computed from the resulting set of nearest-neighbor distances. Comparisons to the “ideal” frame-connection CDF *F*_ideal_ were made by subtracting *F*_ideal_ from the binned CDF *F* of the results being compared. The difference *δ* ≡ *F* − *F*_ideal_ provides a visual tool for comparing frame-connection results. A deviation *δ* < 0 suggests that localizations were connected that should not have been, since such over-connection would increase the expected nearest-neighbor distance. Similarly, a difference *δ* > 0 suggests that frame-connection did not connect localizations which should have ideally been connected. Although this trend for *δ* may not necessarily hold for exceptionally high localization densities (e.g., for very high localization densities, incorrect connections may in fact cause the mean nearest-neighbor distance to decrease), we don’t expect such data to be relevant in SMLM.

## 3 Results

### 3.1 Uniformly Distributed Emitters

**FIGURE 1:**
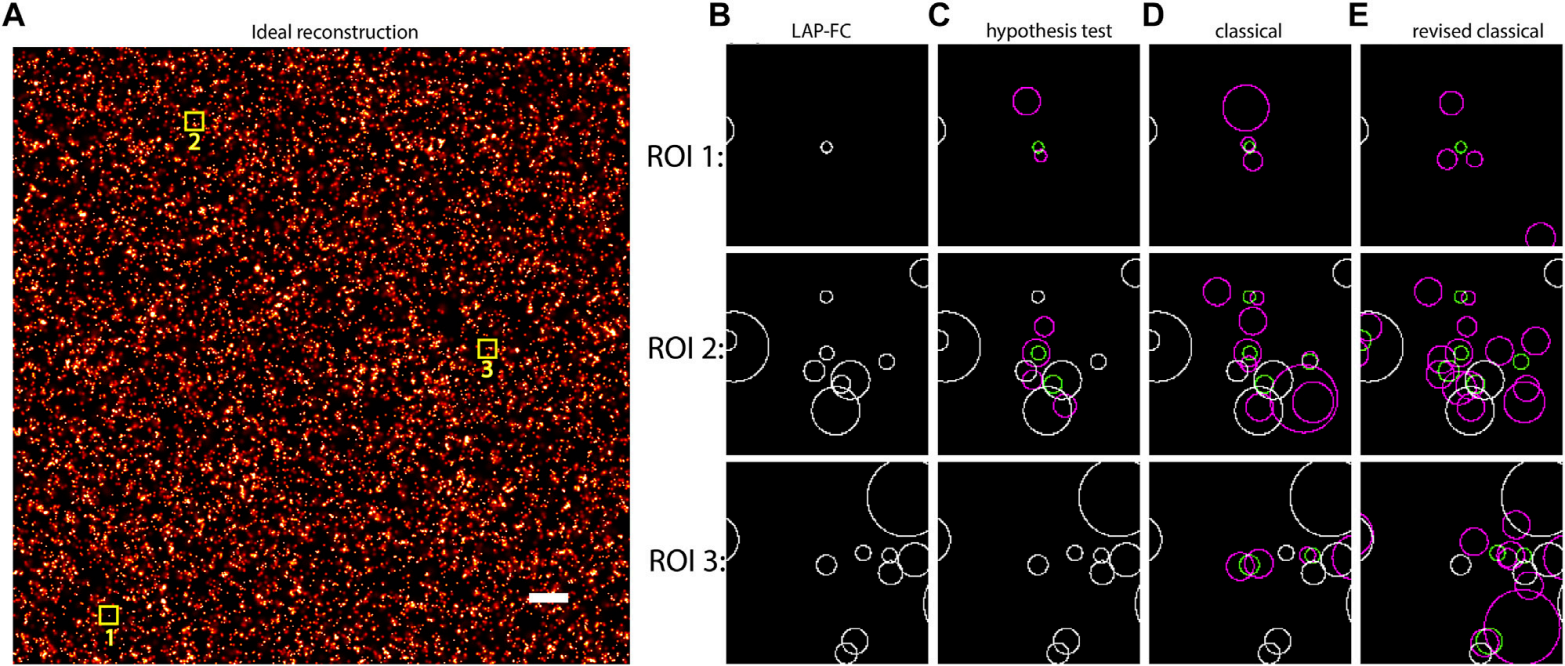
Uniformly distributed emitters with initial density *ρ*_0_ = 10 emitter/pixel^2^, *k*_on_ = 0.005/frame, *k*_off_ = 0.5/frame, *k*_bleach_ = 0.2/frame, *p*_miss_ = 0.01, photon emission rate of 1,000 photons/frame, additional Gaussian noise with *σ* = 0.05 pixels, and 10,000 total frames. **(A)** “Ideal” frame-connection solution, scale bar = 2 pixels. **(B)**–**(E)** Results for the three sub-ROIs marked with yellow squares in **(A)** using **(B)** LAP-FC with a maximum pre-clustering frame gap of five frames and a maximum separation of 5 times the localization error; **(C)** the hypothesis test algorithm with a maximum frame gap of five frames, a maximum separation of 1 pixel, and a level-of-significance of 0.05; **(D)** the classical algorithm with a maximum frame gap of 1 frame and a maximum separation of 0.2 pixels; and **(E)** the revised classical algorithm with a maximum frame gap of five frames and a maximum separation of 2 times the localization error. The circles in **(B)**–**(E)** are centered at the localization position with radii equal to the localization error. Green circles represent localizations from the ideal results from **(A)**. Magenta circles represent the results of the frame-connection algorithm. White circles correspond to frame-connection results matching the ideal results.

Simulated SMLM data for uniformly distributed emitters was generated as described in section 2.4. The frame-connection results from each of the algorithms (LAP-FC, hypothesis test, classical, and revised classical) are shown in Figure 1. ROI selections were made to highlight the performance of LAP-FC in comparison to the other algorithms. For the sole emitter blink present in Figure 1 ROI 1, the LAP-FC algorithm was the only method to completely connect all observed localizations. For Figure 1 ROI 2, the hypothesis test, classical, and revised classical algorithms correctly connected most of the localizations. For Figure 1 ROI 3, the classical and revised classical algorithms again connected most localizations correctly while failing to connect some others.

**FIGURE 2:**
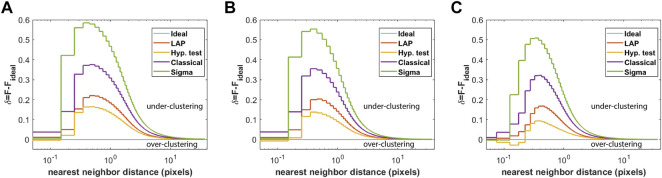
Deviation of the nearest-neighbor distance CDF from that of the ideal frame-connection result ensembled over multiple uniform emitter simulations, where nearest-neighbors are restricted to appear within five frames of one another. The green line corresponds to the revised classical method, the purple line to the classical method, the yellow line to the hypothesis test method, the red-orange line to the LAP-FC method, and the blue line is the *δ* = 0 baseline. The number of simulations and initial densities were varied as **(A)** 40 simulations with *ρ*_0_ = 5 emitters/pixel^2^, **(B)** 20 simulations with *ρ*_0_ = 10 emitters/pixel^2^, and **(C)** 10 simulations with *ρ*_0_ = 20 emitters/pixel^2^.

To compare the frame-connection algorithms for data with varying densities, a total of 40, 20, and 10 independent uniform emitter simulations were generated for initial emitter densities of *ρ*_0_ = 5 emitters/pixel^2^, *ρ*_0_ = 10 emitters/pixel^2^, and *ρ*_0_ = 20 emitters/pixel^2^, respectively, with the parameters otherwise matching those described in section 2.4. The deviation *δ* of the nearest-neighbor distance CDF from the ideal CDF was computed as described in section 2.5. The results are shown in Figure 2. For a relatively low initial emitter density of 5 emitters/pixel^2^, all of the algorithms tend to under-cluster localizations, with LAP-FC showing closer correspondence to the ideal case; however, the hypothesis-test may slightly over-cluster as indicated by the dip of *δ* < 0 in Figure 2A. For an initial density of 10 emitters/pixel^2^, the hypothesis test algorithm shows the closest correspondence to the ideal frame-connection results, however the dip *δ* < 0 seen in Figure 2B suggestive of over-clustering is more prominent than in Figure 2A. The LAP-FC algorithm shows the closest correspondence to the ideal result without indication of over-clustering. For an initial emitter density of 20 emitters/pixel^2^, Figure 2C suggests that the hypothesis testing method is largely over-clustering localizations. The LAP-FC algorithm otherwise shows the closest correspondence to the ideal frame-connection results without significant over-clustering, however a small dip of *δ* < 0 was present at a scale not visible in the figure.

For the simulations described in the preceding paragraph, histograms of the durations of frame-connected localizations were generated and are shown in Supplementary Figure S2. Comparing to the expected distribution (geometric with probability *p* = 1 − exp(−*k*), where *k* ≡ *k*_off_ + *k*_bleach_) of frame-connected durations, all methods appear to have an over-abundance of short durations, with the trend being similar at each tested density. LAP-FC and the hypothesis test method more closely reproduce the expected distribution than the classical and revised classical methods, with the hypothesis test showing the closest correspondence.

To test the robustness of LAP-FC with respect to its estimates of *k*_on_, *k*_off_, *k*_bleach_, and *p*_miss_, LAP-FC was repeated for the 20 *ρ*_0_ = 10 emitters/pixel^2^ simulations described above with varying values of each parameter. For each of the 20 simulations, LAP-FC was applied and the internally estimated values ${\hat{k}}_{\text{on}}$ , ${\hat{k}}_{\text{off}}$ , ${\hat{k}}_{\text{bleach}}$ , and ${\hat{p}}_{\text{miss}}$ were noted (see Supplementary Table S1). LAP-FC was then applied to each simulation with externally prescribed values of *k*_on_, *k*_off_, *k*_bleach_, and *p*_miss_, with each parameter being varied individually with the other parameters held fixed at their true simulated value. Each parameter was varied to their upper and lower bound (see Supplementary Text 1) as well as to the maximum and minimum values estimated in the original LAP-FC application described above. The resulting values of *δ* were computed as described in 2.5 and plotted in Supplementary Figure S1. According to the results in Supplementary Figure S1, even large deviations in parameter estimates from the true values rarely lead to over-clustering by LAP-FC, and in all observed cases (i.e., excluding the upper and lower bound demonstrations), the results show little deviation from those when all parameters are set to their simulated value.

### 3.2 Simulated Dimer Emitters

**FIGURE 3:**
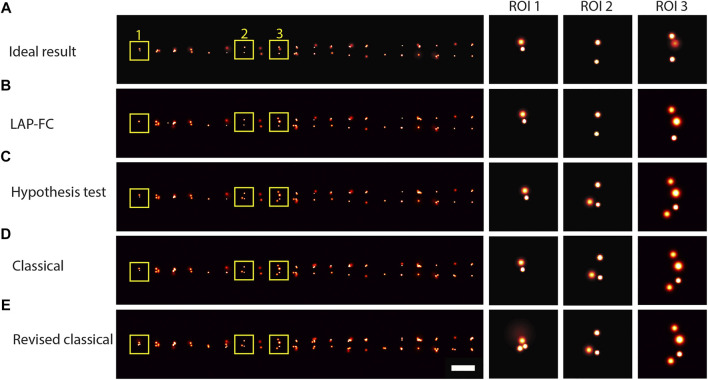
Gaussian reconstruction images for frame-connection results on simulated dimer emitters with spatial separations ranging from 0.1-1 pixel, *k*_on_ = 0.005/frame, *k*_off_ = 0.5/frame, *k*_bleach_ = 0.2/frame, *p*_miss_ = 0.01, photon emission rate of 1,000 photons/frame, and 10,000 total frames. **(A)** “Ideal” frame-connection solution, scale bar = 2 pixels. **(B)**–**(E)** Results for the three sub-ROIs marked with yellow squares in **(A)** using **(B)** LAP-FC with a maximum pre-clustering frame gap of five frames and a maximum separation of 5 times the localization error; **(C)** the hypothesis test algorithm with a maximum frame gap of five frames, a maximum separation of 1 pixel, and a level-of-significance of 0.05; **(D)** the classical algorithm with a maximum frame gap of 1 frame and a maximum separation of 0.2 pixels; and **(E)** the revised classical algorithm with a maximum frame gap of five frames and a maximum separation of 2 times the localization error. Scale bar = 2 pixels.

Dimerized emitters were simulated at 20 spatial separations ranging uniformly from 0.1-1 pixel to investigate frame-connection performance for closely spaced emitters. Gaussian reconstruction images are shown for the results of each of the frame-connection algorithms in Figure 3. Overall, each of the tested algorithms performed well enough to observe the general trend in the data clear from the ideal result in Figure 3A (that is, pairs of closely spaced emitters with an increasing pair separation from left to right). The classical and the revised classical methods (Figure 3D,E, respectively) did not correctly connect as many localizations as the LAP-FC and hypothesis test methods (Figure 3B,C, respectively), however no over-clustering was apparent. Overall, the LAP-FC performed better than the other methods tested. No apparent over-clustering artifacts were introduced by any of the four algorithms tested.

### 3.3 High Duty Cycle Actin With Multi-Emitter Fitting

**FIGURE 4:**
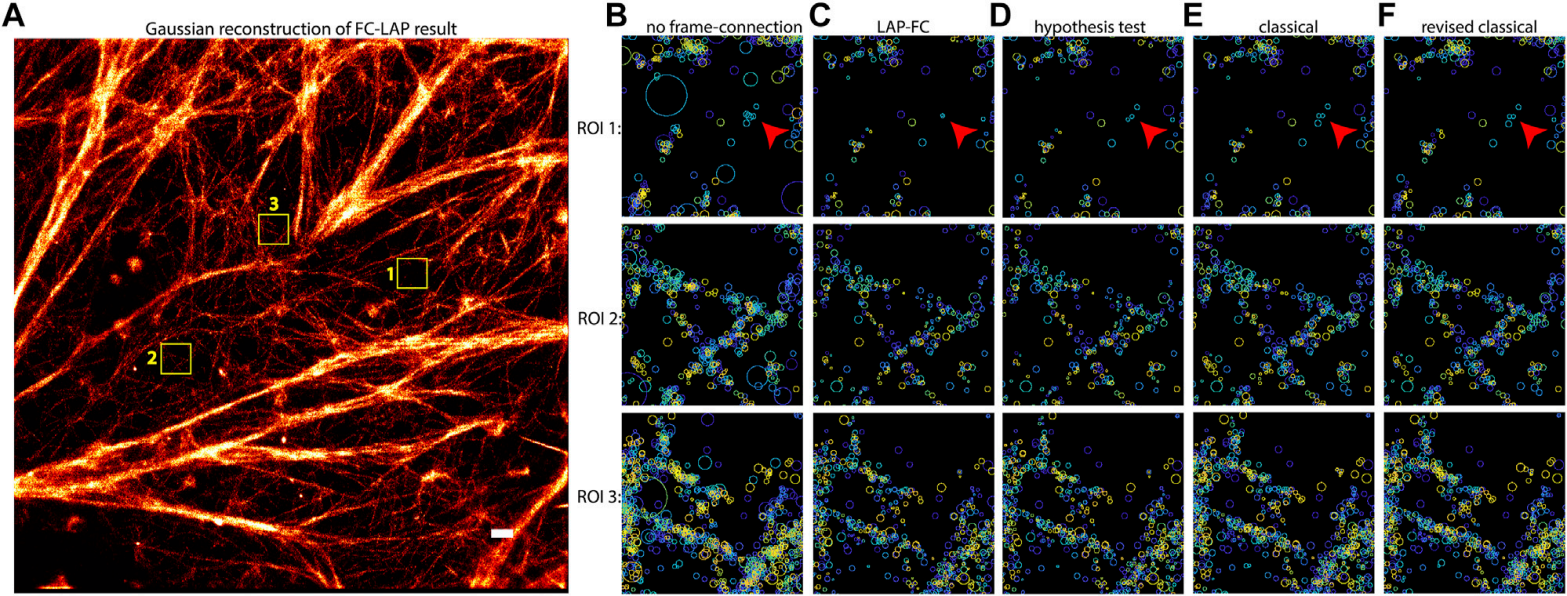
Frame-connection results for actin microfilament localizations generated by multi-emitter fitting. **(A)** Gaussian reconstruction image of the LAP-FC frame-connection results. Selected ROIs indicated by numbered yellow boxes in (A) are shown for the frame-connection results using **(C)** LAP-FC with a maximum pre-clustering frame gap of five frames and a maximum separation of 5 times the localization error; **(D)** the hypothesis test algorithm with a maximum frame gap of five frames, a maximum separation of 1 pixel, and a level-of-significance of 0.05; **(E)** the classical algorithm with a maximum frame gap of 1 frame and a maximum separation of 0.2 pixels; and **(F)** the revised classical algorithm with a maximum frame gap of five frames and a maximum separation of 2 times the localization error. Scale bar = 0.5 μm. Localizations in **(B)**–**(F)** are displayed as circles of radius equal to the localization error centered on the estimated position and color-coded to indicate time, with dark blue indicating the start of the experiment and yellow indicating the end of the experiment. The red arrows in **(B)**–**(F)** ROI 1 point to a qualitatively interesting set of localizations.

An SMLM dataset resulting from Bayesian multi-emitter fitting (Fazel et al., 2019a) of actin data with a relatively high localization density was used to compare the performance of the tested frame-connection algorithms. The results are shown in Figure 4. Inspecting the ROI selections made in Figures 4C–F and comparing to the non-frame connected results in Figure 4B, each of the algorithms appear to make reasonable connections based on localization spatiotemporal proximity. The LAP-FC and hypothesis testing algorithms made the most connections as is noticeable by the feature sharpness in Figures 4C,D. The classical and revised classical methods both fail to connect a pair of relatively isolated nearby emitters on the right hand side of ROI 1 (shown as blue circles and pointed to by small red arrows), which when compared to Figure 4B seem to be arising from the same emitter.

## 4 Discussion

SMLM is rapidly becoming a commonplace tool for researchers in need of nanoscale spatial resolution in fluorescence microscopy. The expansion of SMLM outside of dedicated research labs necessitates reliable analyses which can be trusted without expert intervention. Quantitative analysis of the resulting super-resolved localizations requires, in many cases, a well-characterized correspondence between localizations and emitters. That is to say, many analyses of super-resolved localizations require a one-to-one relationship between localization and emitter. While recent techniques have largely solved this localization clustering problem (Fazel et al., 2019b), any such method will be limited by the reliability of the input localizations. If the input localizations contain a very large proportion of repeated localizations, such post-processing tools may be pushed to their practical limits, for example leading to infeasible computational costs. Alternatively, localizations which have been over-clustered (i.e., localizations from distinct emitters that were connected together) represent a loss of information unlikely to be captured by any post-processing analysis.

Many steps in SMLM data analysis have been refined and validated (e.g., fitting the localizations and determining the error in their positions), however the frame-connection problem has received little attention. Known existing methods for solving the frame-connection problem have not reached an optimal solution. We have shown that the classical and the revised classical methods are perhaps too conservative in their assignment of connections to make optimal use of the data. On the other hand, the hypothesis testing method is perhaps too liberal in its assignment of connections. We have shown that the hypothesis testing method for frame-connection, which typically provides more appealing results than the classical and revised classical methods, is susceptible to over-clustering at high densities. Furthermore, results of the classical, revised classical, and the hypothesis test algorithms rely heavily on the selection of arbitrary thresholds. We have shown that, by formulating the frame-connection problem as a linear assignment problem with statistically motivated assignment costs, these common artifacts can largely be reduced, with the added benefit that arbitrary thresholds are used only in a pre-processing step. Our algorithm accounts for the local emitter densities, the kinetic rates of blinking, and the possibility of missing localizations of a visible emitter. By combining all of this knowledge, our algorithm exceeds the performance of other known frame-connection problems with minimal to no over-clustering.

## Data Availability

The raw data supporting the conclusions of this article will be made available by the authors, without undue reservation.
