# Corrigendum: Spatiotemporal Clustering of Repeated Super-Resolution Localizations via Linear Assignment Problem

**DOI:** 10.3389/fbinf.2021.836606

**Published:** 2022-01-25

**Authors:** David J. Schodt, Keith A. Lidke

**Affiliations:** Department of Physics and Astronomy, University of New Mexico, Albuquerque, NM, United States

**Keywords:** fluorescence microscopy, super-resolution, image analysis, computational modeling, single molecule techniques

In the original article, there was an error. Eq. (2) was written incorrectly. A correction has been made to *MATERIALS AND METHODS*, *Estimating Local Emitter Densities and Kinetic Rates*, *Paragraph Number 2*:

The expected cumulative number of localizations observed by frame *f* is given by

$$\begin{array}{l} \left\langle n_{\text{cumulative}}\right\rangle \left(f\right)\approx N_{\text{emitters}}\left(1-p_{\text{miss}}\right)\tau \left\{\frac{1-\exp\left[-\lambda_{1}\left(f-1\right)\right]}{\lambda_{1}}-\frac{1-\exp\left[-\lambda_{2}\left(f-1\right)\right]}{\lambda_{2}}\right\} \end{array}$$ (2)

In the original article, there was an error. An unlabeled equation was written incorrectly. Additionally, a line of text in the associated paragraph was written incorrectly. A correction has been made to *MATERIALS AND METHODS*, *Estimating Local Emitter Densities and Kinetic Rates*, *Paragraph Number 4*:

The local pre-cluster density corresponding to each pre-cluster is estimated by finding the *k* (chosen to be two in this study) nearest pre-clusters and then computing $\rho_{c}=\left(k+1\right)/\pi r_{k}^{2}$ where *r*_*k*_ is the distance to the *k*th nearest pre-cluster. The underlying local emitter density present at the beginning of the experiment is then estimated for each pre-cluster as

$${\hat{\rho }}_{0,\text{local}}=\rho_{c}\frac{1}{{\hat{k}}_{\text{off}}\hat{\tau }}\frac{1}{1-{\hat{p}}_{\text{miss}}}{\left\{\frac{1-\exp\left[-{\hat{\lambda }}_{1}\left(f_{\text{end}}-1\right)\right]}{{\hat{\lambda }}_{1}}-\frac{1-\exp\left[-{\hat{\lambda }}_{2}\left(f_{\text{end}}-1\right)\right]}{{\hat{\lambda }}_{2}}\right\}}^{-1}$$

where *f*_end_ is the last frame containing localizations in the experiment.

The authors apologize for this error and state that this does not change the scientific conclusion of the article in any way. The original article has been updated.

