# Supplementary material for: Spatiotemporal Clustering of Repeated Super-Resolution Localizations via Linear Assignment Problem
Source: Front Bioinform. 2021 Oct 20;1:724325. doi: 10.3389/fbinf.2021.724325 (PMC9581011; doi:10.3389/fbinf.2021.724325)
Supplement: Supplementary file 1 [file DataSheet1.PDF]

Supplementary Material

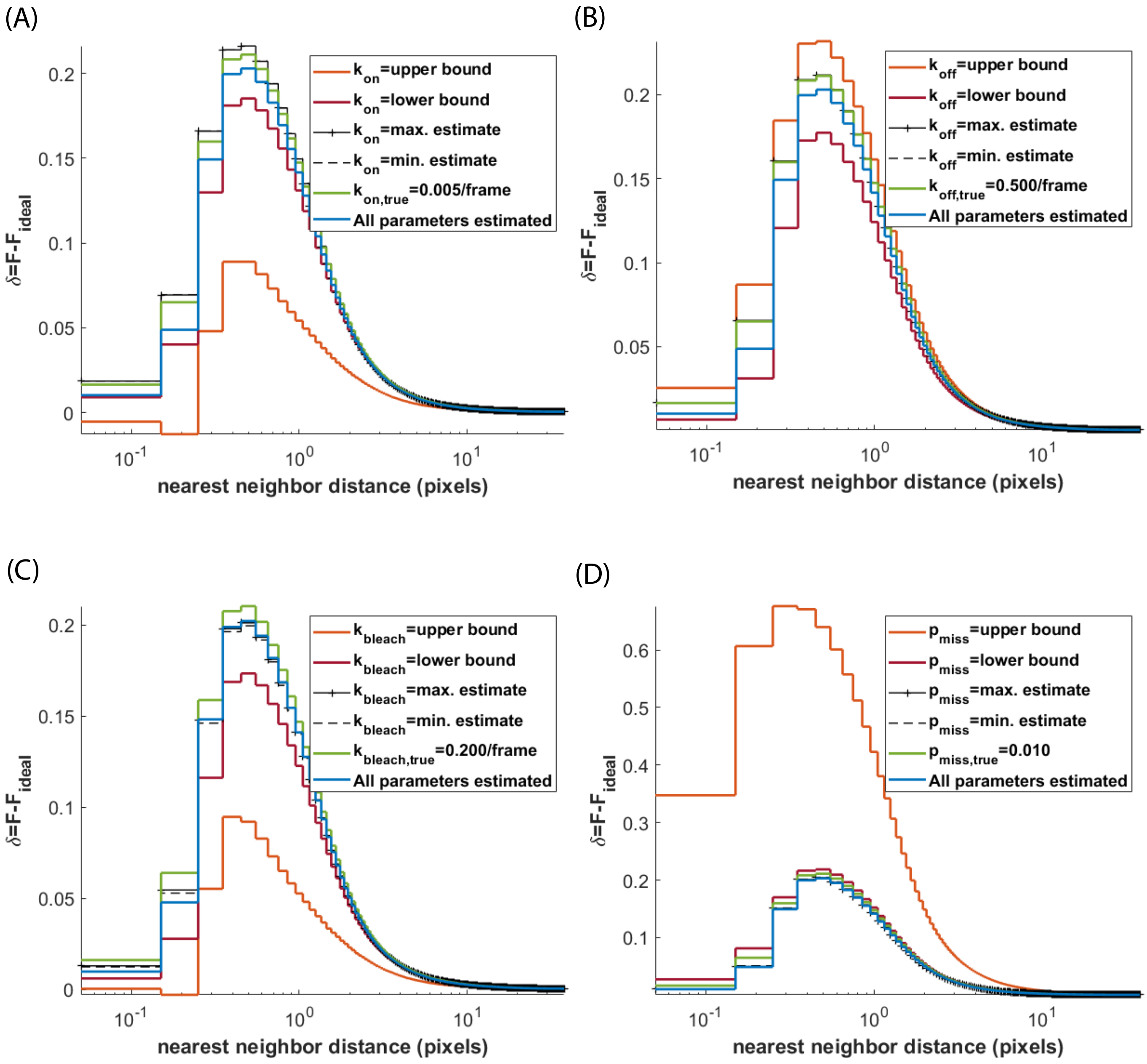

Figure S1. Deviation of the nearest-neighbor distance CDF from that of the ideal frame-connection result for the ensembled simulation of uniform emitters with initial density $\rho_0 = 10$ emitter/pixel^2 shown in figure 2B with varying values of some estimated parameters. The non-varying parameters are set to their true simulated values, except in the results labeled ”All parameters estimated”, for which the algorithm was run exactly as described in the text with no additional parameter restrictions. **(A)** Varying k_{on}. **(B)** Varying k_{off}. **(C)** Varying k_{bleach}. **(D)** Varying p_{miss}.

Table S1. Summary of parameter ranges used in figure S1. The lower and upper bounds are the inherent/enforced constraints on parameters made during parameter estimation (see Supplementary text 1). Min. and max. estimates represent the minimum and maximum values observed over the 20 simulations of uniformly distributed emitters with initial density ρ_0 = 10 emitters/pixel^2 described in the main text. All rate parameters are shown in units of frames^{-1}.

Parameter	Simulated	Lower bound	Upper bound	Min. estimate	Max. estimate
k_{on}	0.005	10^{-5}	41,617/10,000	0.011	0.012
k_{off}	0.500	1/41,617	1	0.503	0.514
k_{bleach}	0.200	10^{-5}	∞	0.121	0.131
p_{miss}	0.010	0	1-1/10,000	0.030	0.034

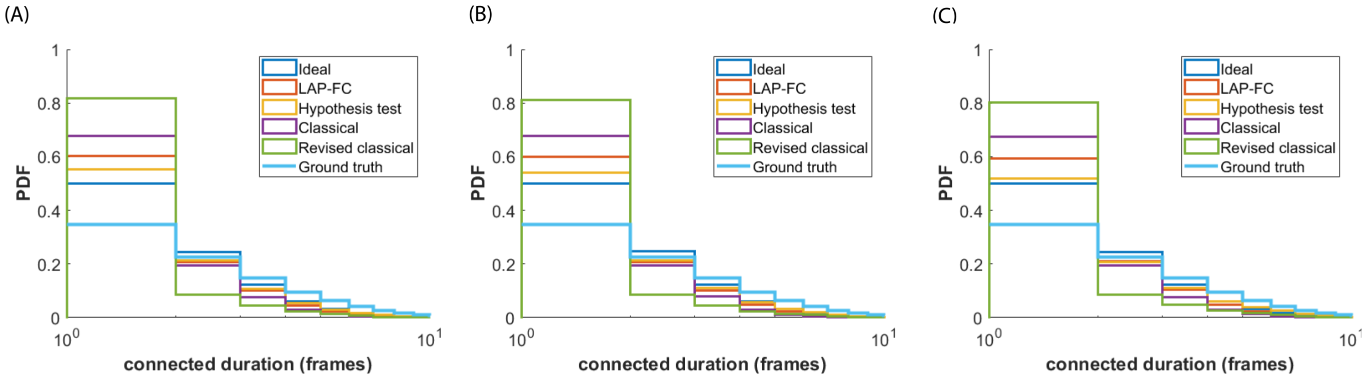

Figure S2. Histograms of the recovered durations of frame-connected localizations for the simulations shown in figure 2. The number of simulations and initial densities were varied as **(A)** 40 simulations with ρ_0 = 5 emitters/pixel^2, **(B)** 20 simulations with ρ_0 = 10 emitters/pixel^2, and **(C)** 10 simulations with ρ_0 = 20 emitters/pixel^2.

1 SUPPLEMENTARY TEXT 1: DESCRIPTION OF NUMERICAL PARAMETER ESTIMATION

As described in the main text, the off rate k_{off} is approximated as $\hat{k}_{\text{off}} = n_{\text{clusters}}/n$ where n_{clusters} is the total number of pre-clusters and n is the total number of localizations. As such, $\hat{k}_{\text{off}}$ is intrinsically bounded to the interval $[n^{-1}, 1]$. The sum of $k_{\text{off}} + k_{\text{bleach}}$ is estimated from the mean cluster duration as $\widehat{k_{\text{off}} + k_{\text{bleach}}} = -\log\left(1 - 1/\bar{N}\right)$. The bleaching rate k_{bleach} is estimated as $\hat{k}_{\text{bleach}} = \max(10^{-5}, \widehat{k_{\text{off}} + k_{\text{bleach}}} - \hat{k}_{\text{off}})$, thus $\hat{k}_{\text{bleach}}$ is bounded to the interval $[10^{-5}, \infty]$. Enforcing the lower bound for k_{bleach} was found to be necessary when photobleaching is negligible (e.g., for DNA-PAINT data), in which case the difference $\widehat{k_{\text{off}} + k_{\text{bleach}}} - \hat{k}_{\text{off}}$ may be a (nearly 0) negative number.

The estimate for p_{miss}, $\hat{p}_{\text{miss}} = 1 - \overline{n_c/N}$, is intrinsically bounded to the interval $[0, 1 - f_{\text{end}}^{-1}]$.

Once k_{off}, k_{bleach}, and p_{miss} have been estimated, we can estimate k_{on} and N_{emitters} by fitting a model to the cumulative number of localizations (as stated in the main text)

$$n_{\text{cumulative}}(N_{\text{emitters}}, k_{\text{on}}) \approx N_{\text{emitters}}(1 - \hat{p}_{\text{miss}}) \frac{k_{\text{on}}}{k_{\text{on}} + \widehat{k_{\text{off}} + k_{\text{bleach}}}} \Big\{ \frac{1}{\lambda_1(k_{\text{on}})} \exp[-\lambda_1(k_{\text{on}})(f-1)] - \frac{1}{\lambda_2(k_{\text{on}})} \exp[-\lambda_2(k_{\text{on}})(f-1)] \Big\}$$

subject to the following constraints:

$$k_{\text{on}} \in \left[10^{-5}, \frac{n}{f_{\text{end}}}\right]$$

$$N_{\text{emitters}} \in \left[\max_f n(f), n_{\text{clusters}}\right]$$

where $\max_f n(f)$ is the maximum number of localizations observed in any one frame of the data. Initial values of $k_{\text{on}} = f_{\text{end}}^{-1}$ and $N_{\text{emitters}} = k_{\text{bleach}} n_{\text{clusters}}$ were found to work well in all simulations tested. If the initial value of $N_{\text{emitters}} = k_{\text{bleach}} n_{\text{clusters}}$ falls outside of the constraint interval, the initial value for N_{emitters} is instead chosen to be the midpoint of the constraint interval.
